# New Therapies to Correct the Cystic Fibrosis Basic Defect

**DOI:** 10.3390/ijms22126193

**Published:** 2021-06-08

**Authors:** Christelle Bergeron, André M. Cantin

**Affiliations:** 1 Department of Medicine, Respiratory Division, Faculty of Medicine and Health Sciences, University of Sherbrooke, Sherbrooke, QC J1H 5N4, Canada; Christelle.Bergeron@USherbrooke.ca; 2 Centre de Recherche du Centre Hospitalier Universitaire de Sherbrooke, Respiratory Division, Faculty of Medicine, University of Sherbrooke, 3001, 12e Avenue Nord, Sherbrooke, QC J1H 5N4, Canada

**Keywords:** cystic fibrosis, CFTR, CFTR modulators, gene therapy

## Abstract

Rare diseases affect 400 million individuals worldwide and cause significant morbidity and mortality. Finding solutions for rare diseases can be very challenging for physicians and researchers. Cystic fibrosis (CF), a genetic, autosomal recessive, multisystemic, life-limiting disease does not escape this sad reality. Despite phenomenal progress in our understanding of this disease, treatment remains difficult. Until recently, therapies for CF individuals were focused on symptom management. The discovery of the *cystic fibrosis transmembrane conductance regulator* (*CFTR*) gene and its product, a protein present at the apical surface of epithelial cells regulating ion transport, allowed the scientific community to learn about the basic defect in CF and to study potential therapies targeting the dysfunctional protein. In the past few years, promising therapies with the goal to restore CFTR function became available and changed the lives of several CF patients. These medications, called CFTR modulators, aim to correct, potentialize, stabilize or amplify CFTR function. Furthermore, research is ongoing to develop other targeted therapies that could be more efficient and benefit a larger proportion of the CF community. The purpose of this review is to summarize our current knowledge of CF genetics and therapies restoring CFTR function, particularly CFTR modulators and gene therapy.

## 1. Introduction

**Figure 1:**
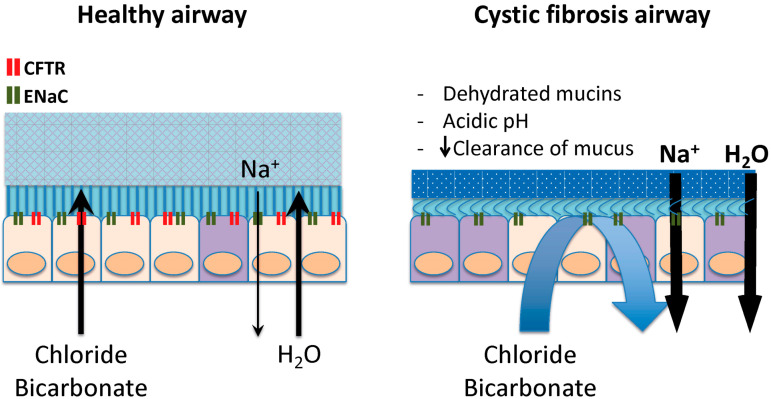
Role of the CFTR in the regulation of mucus viscosity and pH at the epithelial surface. Healthy mucus is composed primarily of mucins and water. Hydration and pH regulate mucus viscosity, and both of these functions are controlled by CFTR at the apical surface of epithelial cells. The movement of chloride dictates the degree to which mucus retains water whereas CFTR-mediated bicarbonate flux plays a key role in defining pH which is critical to healthy anti-bacterial response. In the absence of CFTR, secretions are viscous, adhere to mucosal surface and obstruct cylindrical structures such as small airways and sub-mucosal glands. The acidic pH further contributes to decrease host anti-bacterial defenses.

Cystic fibrosis (CF) has evolved from a fatal disease of unknown cause to a condition we understand with molecular precision [1]. In the absence of CFTR, the most important regulator of airway surface liquid hydration is lost (Figure 1), mucociliary clearance is suppressed, airway pathogens proliferate and toxic neutrophil mediators are released, causing bronchial destruction known as bronchiectasis [2]. Promising new therapies aimed at restoring CFTR function are rapidly transforming the lives of most individuals with CF [3] and are the object of the current review. Other novel therapies being developed but not discussed in this review include mucociliary clearance modulators, anti-inflammatories and anti-infective drugs [4].

### Classification of CFTR Defects: An Essential Step in CF Drug Discovery

Over 2000 variants of the *CFTR* gene have been reported, of which at least 360 are CF-causing variants which will be referred to in this review as mutations [5]. The molecular and clinical consequences of bearing a combination of two homozygous or heterozygous *CFTR* mutations are highly variable and pose a major challenge for therapeutic development. Classification of mutations based on the impact they have on protein expression and function has greatly facilitated high throughput screening of compounds and CFTR-directed drug development [6,7,8]. However, it is important to note that even with the same genotype, a variable response to therapy is frequent and poses a further challenge to therapeutic development.

**Figure 2:**
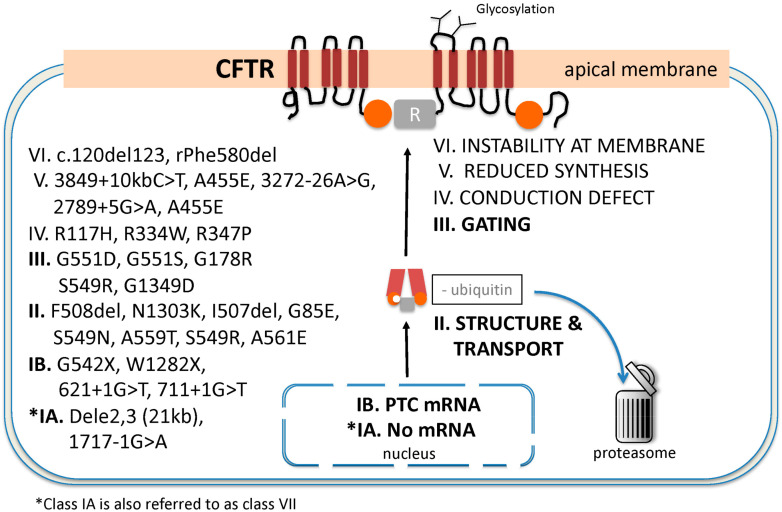
Classification of the *CFTR* disease-causing mutations. Classes I–III comprise most of the mutations associated with classical CF disease. Examples of alleles of each class are listed for each mutation class. * Class 1A is often referred to as class VII as originally suggested by De Boeck and Amaral [6].

The classification of *CFTR* mutations is now most often divided into seven groups from class I through VII as described by Kris De Boeck and Margarida Amaral [6]. Others have suggested that class VII be renamed class I subtype A (IA) since no mRNA is produced and the clinical manifestations of disease are severe as is the case for mutations of classes I to III. For the purpose of this review, we have adopted the latter classification using classes IA and B to VI (Figure 2). The frequency of allele occurrence in CF varies greatly among racial and ethnic groups [9]. Severe manifestations of typical CF disease are generally associated with mutations of classes I-III whereas disease manifestations in other classes are usually less severe [10]. However, several *CFTR* mutations have characteristics that cross multiple classes, rendering such classifications necessarily imperfect [11]. Therapeutic development has largely focused on ameliorating CTFR function associated with *CFTR* gene defects within classes I–III.

Class I includes frameshift, splicing and non-sense mutations that result in incomplete or absent mRNA and no functional CFTR protein synthesis. Class IA includes large deletion mutations with no mRNA synthesis [6,8]. Class IB includes mostly non-sense mutations resulting in a premature termination codon and the production of unstable mRNA. The abnormal mRNA is rapidly recognized and eliminated by the non-sense mRNA decay (NMD) surveillance system [12,13]. CFTR protein fragments synthesized by class IB mutations are recognized and degraded in the proteasome before they can be inserted into the apical membrane.

Class II mutations result in misfolded CFTR protein. The CFTR structural defect is recognized by the cell’s quality-control system, which will ubiquitinate the abnormal protein in the endoplasmic reticulum and direct it to the proteasome for degradation (a process known as endoplasmic-reticulum-associated degradation or ERAD) before it can be transported to the apical membrane. Abnormal CFTR biogenesis, protein-folding and degradation is regulated by a complex proteostatic network that continues to be the object of intense pharmacological investigation [14]. The most common class II allele is the c.1521_1523delCTT (legacy name F508del) mutation, which is present in 85% of all individuals with classical CF disease worldwide, although the frequency of class II mutations other than F508del varies considerably between populations and is increased in Southern Europe and other regions [15]. The little amount of F508del CFTR protein that makes it to the membrane also has a severe class III gating defect and is unstable at the apical membrane (class VI) [11]. Correction of multiple defects is therefore essential to restore CFTR function in individuals bearing the F508del allele.

Class III mutations involve impaired gating of the CFTR channel. Although the protein is structurally intact and migrates correctly to the apical membrane where it is stable, CFTR proteins with gating mutations have an open probability which is decreased up to 100-fold compared to wild-type CFTR [16]. The gating defect is due to abnormal ATP-binding to the nucleotide binding domains (NBD1 and NBD2) and lack of ATP hydrolysis. The resulting dysfunction of CFTR is severe and causes classical manifestations of CF disease. The most common class III mutation is G551D. Correction of the gating defect with a single drug known as a potentiator is sufficient to partially restore a considerable amount of CFTR function and markedly improve patient outcomes [17].

Class IV mutations cause a conduction defect in which the anion selectivity of CFTR is altered, leading to decreased chloride and bicarbonate conductance. Because partial chloride or bicarbonate conductance is preserved, individuals with class IV mutations will often express milder CF disease [18]. The most common mutation in this class is R117H. This mutation also produces a gating (class III) defect which can be partially rescued by potentiator therapy with ivacaftor [19]. Furthermore, the disease severity of individuals expressing the genotype F508del/R117H varies greatly according to a variable sequence within intron 9 of five, seven or nine thymidine bases (poly-T repeats), which increases the risk of exon-10 (formerly named exon 9) skipping [20,21]. The shorter the poly-T repeat, the more severe are CF disease manifestations. The R117H mutation illustrates the challenges of using a *CFTR* mutation classification system to predict disease severity and drug responses.

Class V mutations result in greatly reduced amounts of functional CFTR at the apical membrane due to promoter mutations, alternate splicing defects or missense mutations resulting in abnormal mRNA transcripts [20]. The severity of clinical manifestations within this class can vary between patients and within different organs of the same patients but are generally mild.

Class VI mutations produce functional CFTR protein which migrates to the plasma membrane but is unstable [22]. Plasma membrane CFTR endocytosis or turnover is accelerated, thus reducing CFTR density and function [23].

**Figure 3:**
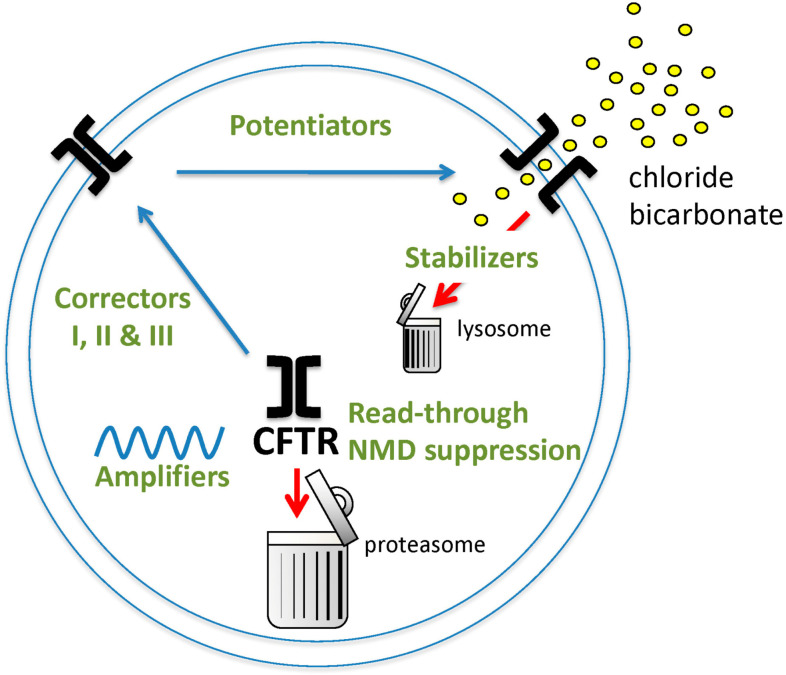
Pharmacological strategies for restoring CFTR function in CF individuals with various classes of *CFTR* mutations. Illustrated in green are agents that increase the open probability of CFTR (potentiators), facilitate escape of misfolded protein from ERAD (correctors of class I act on NBD1, class II on NDBII and class III have an additive corrector effect in the presence of class I corrector), increase the amount of CFTR mRNA (amplifiers), increase proper translation of mRNA with PTC mutations (read-through agents), decrease NMD (NMD suppressor) and prevent degradation of CFTR protein inserted in the plasma membrane (stabilizers).

Promising strategies to correct the function of the abnormal CFTR protein resulting from different mutations are summarized in Figure 3. Classes of CFTR modulating agents include potentiators, correctors, amplifiers, read-through agents, NMD suppressors and stabilizers.

## 2. CFTR Modulators

### 2.1. First-Generation CFTR Modulators

#### 2.1.1. Ivacaftor

Ivacaftor (VX-770, trade name Kalydeco, Vertex Pharmaceuticals) is the first CFTR modulator approved for clinical use in CF patients aged 6 and above by the Food and Drug Administration (FDA). It was approved following pooled safety and efficacy data from clinical trials in 353 CF patients aged 6–53 years and with a G551D mutation (*n* = 213) or who were homozygous for F508del. Patients received 150 mg of ivacaftor orally twice daily (*n* = 221) or placebo (*n* = 132) for 16–48 weeks. No clinically significant improvement was observed in the F508del homozygous population [24]. Major improvements were observed in persons with CF and the G551D-*CFTR* mutation for several CF disease parameters including a decrease in sweat chloride concentration to values approximating normal values, an average increase in the percent predicted forced expiratory volume in one second (ppFEV_1_) of 10%, significant weight gain, particularly in children and a 55% decrease in the frequency of respiratory exacerbations [17,25,26]. Such clinical improvements were unheard of with all other treatments prior to ivacaftor. This was the first time a therapy directed at correcting the basic defect causing CF was successful, thus providing immeasurable hope for all persons with CF. Although relatively few individuals with CF carry the G551D mutation (2–5%) or other gating mutations, the results provided evidence for the first time that partial restoration of CFTR function could improve, and even reverse CF disease manifestations previously thought to be irreversible.

Subsequent studies have determined that the benefits of ivacaftor therapy are sustained with a significant decrease in positive sputum cultures for *Pseudomonas aeruginosa* [27,28,29,30]. Although ivacaftor therapy does not eradicate *P. aeruginosa* in persons with CF and G551D [31], it increases the taxonomic richness of airway bacteria and decreases airway inflammation [28,32]. Recently, analyses of data collected through national CF registries in the United States, United Kingdom and Ireland have confirmed the long-term benefits of ivacaftor on lung function, airway microbiology, the number of hospitalizations and antibiotic use [30,33,34]. Furthermore, ivacaftor use is safe and beneficial even in CF patients with G551D and severe lung disease [35,36,37].

One of the challenges of assessing new therapies for CF is to detect clinically meaningful changes in children too young to perform conventional forced spirometry. Studies using gamma scintigraphy after inhalation of technetium-99m-labeled sulfur colloid particles have shown that ivacaftor therapy in CF patients with the G551D-*CFTR* mutation improves mucociliary clearance (MCC) which correlates with improved lung function. A surrogate measure for MCC that does not require the use of radioactive material is the lung clearance index (LCI) [38]. The LCI is a nitrogen gas multiple breath washout technique that can be used in young children. When used in centers with expertise and proper equipment, LCI provides clinically meaningful and reproducible information to monitor structural airway changes and lung function [39]. Ivacaftor has been shown to improve the LCI in CF patients with G551D and other gating mutations, even in patients under 5 years old [40].

Imaging with chest computed tomography scan (CT) has confirmed that ivacaftor in individuals with CF and a G551D mutation can improve mucus plug clearance from the airways and durably decrease structural anomalies such as peri-bronchial thickening [41,42,43]. Improvements in CT imaging of sinuses have also been reported, further illustrating the efficacy of ivacaftor in restoring mucociliary clearance [44].

Ivacaftor also corrects intestinal pH and improves the nutritional status of patients with gating mutations [27]. Remarkably, ivacaftor reverses pancreatic insufficiency in young as well as older patients [45,46]. Many individuals with the G551D-*CFTR* mutation taking ivacaftor normalize their fecal elastase levels and decrease or suspend pancreatic enzyme replacement therapy. In contrast to the rapid improvements with ivacaftor treatment observed in the function of other organs, restoration of pancreatic sufficiency may occur years after starting therapy [47,48,49]. Furthermore, ivacaftor can resolve bouts of chronic pancreatitis [50], although ivacaftor restoration of pancreatic enzyme secretion in some CF individuals may be associated with novel bouts of pancreatitis.

In addition to the maldigestion associated with exocrine pancreatic insufficiency, glucose intolerance is observed in approximately one third of patients 10 years and older, and CF-related diabetes (CFRD) is present in 13% of children at screening by oral glucose tolerance testing [33,51,52]. The incidence of CFRD increases with age and could reach 40% in the adult population. CFRD is associated with a much greater treatment burden and a more rapid decline of lung function, thus highlighting the negative health impacts of CFRD. Individuals with CFRD and the G551D-*CFTR* mutation who are treated with ivacaftor often show an improvement in insulin secretion [53], a decrease in insulin use and occasionally a complete resolution of CFRD [54,55], once again illustrating that not all CF-related complications are irreversible.

Ivacaftor was also shown to markedly improve ppFEV_1_ and decrease antibiotic use in patients with severe CF lung disease who did not have a gating mutation but had a residual function (RF) mutation [56].

Ivacaftor is generally well-tolerated and safe; however, some aspects of therapy require monitoring. First, liver function tests (LFT) must be kept under surveillance, particularly in younger patients. A three-fold increase in LFT over the upper limit of normal was reported in 30% of CF individuals aged 2–5 years receiving ivacaftor [57]. Second, the abrupt withdrawal of ivacaftor for whatever reason has been associated in some patients with lung function deterioration, respiratory failure and death within days of drug interruption [58].

As impressive are the ivacaftor-related health improvements in CF individuals with gating mutations, most CF patients treated with ivacaftor will continue to manifest progressive lung function deterioration and chronic airway infections with occasional exacerbations. Recently, Veit et al. reported that the combination of two drugs with potentiator properties added in vitro to cells bearing several different *CFTR* gating mutations can improve CFTR protein gating function with much better efficacy than monotherapy, restoring 70–120% of wt-CFTR function [59].

The arrival of ivacaftor also brought to light the challenges of approving new therapies for individuals with rare mutations in a rare disease. As evidence mounted indicating that in vitro assays can reliably predict drug efficacy in individual patients, the FDA proposed an alternative path instead of the usual clinical trial to predict clinical efficacy of CFTR modulators in patients with rare *CFTR* mutations. In vitro data from rigorously controlled assays in Fisher rat thyroid (FRT) cells expressing rare *CFTR* mutations has become an important step in meeting regulatory requirements for approval of drugs that could help individuals with CF-causing gating mutations not addressed in clinical trials [60]. Although useful for predicting response to potentiator therapy for class III mutations, the assay of CFTR function in FRT cells has significant limitations for class II mutations in which the defective protein is not already in the apical membrane. Small molecules that rescue misprocessed CFTR protein in the FRT assay can be ineffective to restore cAMP in airway epithelial cells, a property that may be related to the absence of important protein-protein interactions in FRT cells [61].

The pharmacokinetic properties of ivacaftor dictate that it be given twice daily. Other CFTR modulators approved for clinical use in combination with ivacaftor are given once daily. To address this issue, a deuterated form of ivacaftor (VX-561, deutivacaftor) has been developed and is more stable with a 15.9-hour half-life, thus suggesting that deuterated ivacaftor could be dosed once daily [62].

#### 2.1.2. Lumacaftor-Ivacaftor

Ivacaftor brought proof that pharmacological correction of defective CFTR protein, even if partial, can provide dramatic health improvements in selected individuals and reverse what once was thought to be irreversible loss of organ function. However, only a small proportion of CF-causing mutations (5%) are amenable to treatment with potentiator therapy alone and the most common CF-causing mutation, F508del, found in 85–90% of patients, is not responsive to ivacaftor monotherapy [24]. If one is to impact the health of most persons with CF, then restoration of F508del CFTR function is essential. Although F508del *CFTR* mutant results in a gating defect, its major biochemical consequence is a misfolded CFTR protein which rapidly undergoes ERAD [63,64].

The term ‘corrector’ was coined to identify a drug allowing a misfolded protein such as F508del-CFTR to escape ERAD and traffic to the plasma membrane [65]. The first corrector approved for clinical use as a combination therapy with ivacaftor in CF is lumacaftor (VX-809, trade name of the ivacaftor and lumacaftor combination: Orkambi, Vertex Pharmaceuticals). Early clinical studies with lumacaftor in CF individuals with F508del were disappointing, and it became clear that the combination of a corrector and a potentiator would be needed [66]. The combination of lumacaftor with ivacaftor (LUM/IVA) provided the first positive, albeit modestly so, results in clinical trials addressing the CFTR defect in individuals with the most common CF-causing mutation. Individuals with a single F508del and a second minimal function (MF) *CFTR* mutation showed no benefit of LUM/IVA [67]. However, those who were F508del homozygous treated with LUM/IVA improved their FEV_1_ by 2.6–4.0% and reduced the number of respiratory exacerbations by 42% [68,69]. Although the improvement in lung function could not be perceived by most patients, it was statistically significant and confirmed the promise of exploring combinations of correctors and potentiators in CF patients with the F508del mutation.

The combination of lumacaftor and ivacaftor markedly increases the incidence of drug-related adverse events [70]. In addition to the risk of increasing liver enzymes, lumacaftor markedly induces the activity of CYP3A4 metabolizing enzyme, thus creating multiple undesired drug-drug interactions and precluding its use in some individuals requiring multiple therapies, a common occurrence in CF [71]. The lumacaftor induction of liver metabolizing enzymes can contribute to the inactivation of contraceptive medication, antidepressant drugs and azole antifungals. Lumacaftor is also commonly associated with adverse respiratory reactions and occasionally with depression, increased blood pressure and increased creatine kinase. Furthermore, ivacaftor reduces the correction efficacy of lumacaftor in cells expressing F508del-*CFTR* [72]. The sum of these effects suggests that lumacaftor is less than ideal as a platform for combination drug development in CF.

#### 2.1.3. Tezacaftor-Ivacaftor

Tezacaftor (VX-661) is a first-generation corrector with efficacy in cells bearing the F508del-*CFTR* mutation similar to that of lumacaftor [72]. However, in comparison to lumacaftor, tezacaftor clearly has a better pharmacokinetic profile, induces fewer adverse events such as bronchospasm and has fewer drug interactions [73]. Tezacaftor also is not a strong inducer of CYP3A4 [74].

The combination of tezacaftor and ivacaftor (TEZ/IVA, trade name Symdeko, Vertex Pharmaceuticals) was studied in CF individuals with two F508del mutations or one F508del and either one residual or one minimal function *CFTR* mutation. A minimal function (MF) *CFTR* mutation produces either no CFTR protein or protein without ion channel function. Therapy with TEZ/IVA did not improve ppFEV_1_ or reduce the frequency of pulmonary exacerbation in people with one F508del and one MF *CFTR* mutation [75]. Other than a modest improvement in sweat chloride concentration, TEZ/IVA was not superior to ivacaftor alone in individuals with F508del and a gating mutation [76]. However, improvements in ppFEV_1_ and a decrease in pulmonary exacerbations were observed in CF individuals homozygous for F508del or with one F508del and one RF *CFTR* mutation [77,78]. These clinical improvements were of a degree similar to those observed with LUM/IVA studies of individuals homozygous for F508del-*CFTR* [77]. In marked contrast to LUM/IVA, the TEZ/IVA combination induced fewer respiratory adverse events, particularly less bronchospasm, making it an attractive therapeutic alternative for individuals who do not tolerate LUM/IVA [79]. Following these studies, TEZ/IVA has received FDA approval for treatment of CF patients 12 years and older with two F508del mutations or one F508del and one of the residual function (RF) mutations listed in Table 1. Recent phase III trial results indicate that TEZ/IVA is also both effective and safe in CF children aged 6–11 years with two F508del or one F508del and one RF mutation [80,81].

### 2.2. Next-Generation CFTR Modulator Therapy

#### 2.2.1. Elexacaftor–Tezacaftor–Ivacaftor

Elexacaftor (VX-445) and VX-659 are next-generation correctors with CFTR binding sites and mechanisms of action that are clearly different from the first-generation correctors lumacaftor and tezacaftor. Tezacaftor is a class I corrector which stabilizes the interface between NBD1 and membrane spanning domains 1 and 2 (NBD1-MSD1/2). Class II correctors affect NBD2 mutations, whereas elexacaftor is a class III corrector which stabilizes F508del-NBD1 affecting the unfolding trajectory of NBD1 [82,83]. Ninety percent of CF individuals carry at least one copy of the F508del mutation. Although the combination of ivacaftor and one of the first-generation correctors results in some clinical improvement, the impact of therapy is often disappointing and insufficient to transform the lives of most CF patients [84]. Furthermore, individuals with classical CF who are heterozygous for F508del and an MF *CFTR* mutation (approximately 30%) do not show any improvement with either dual CFTR-modulator combination of LUM/IVA or TEZ/IVA [75,85]. A critical breakthrough in CF therapeutic development was the discovery that the addition of either elexacaftor or VX-659 to TEZ/IVA increases mature CFTR and improves chloride ion transport when compared to either drug alone or dual combinations in epithelial cells from MF/F508del and F508del/F508del donors [86,87]. The phase II trial results of elexacaftor and VX-659 with TEZ/IVA in patients with the F508del/F508del or MF-F508del genotypes demonstrated marked reductions in sweat chloride levels that approximated the normal range, unprecedented improvements in lung function as determined by the ppFEV_1_ (ELX/TEZ/IVA 13.8% over placebo in F508del/MF; 11% over TEZ/IVA in F508del/F508del) and clinically significant improvement in CF symptoms as determined by the CFQ-R respiratory domain score at day 29 [86,87].

Pivotal phase III clinical trials of triple therapy (ELX/TEZ/IVA, trade name Trikafta, Vertex Pharmaceuticals) confirmed the phase II study results. Individuals heterozygous for F508del/MF 12 years and older were given either a daily dose of 200 mg of elexacaftor and 100 mg of tezacaftor plus 150 mg of ivacaftor twice daily or placebo for 24 weeks [88]. The absolute increase in ppFEV_1_ was 14.3%, and the rate of pulmonary exacerbations was decreased by 63%. Sweat chloride concentration decreased by 41.8 mmol/L and the CFQ-R respiratory domain score improved by 15 points whereas the minimal clinically significant improvement is four points. Significant weight gain has also been reported which is likely related to both better digestion and absorption associated with restored CFTR function. Remarkably, the triple therapy is particularly well-tolerated. Liver enzyme monitoring is recommended as with ivacaftor, and there have been reports of biliary colic and transient testicular pain, as well as increased fertility in women [89,90,91]. Delayed drug interaction between azithromycin and the TEZ/IVA combination manifested by first degree heart block has also been reported [92].

Results very similar to these have also been observed in a 4-week phase III study of individuals homozygous for the F508del genotype comparing ELX/TEZ/IVA to standard of care with TEZ/IVA [93]. Triple therapy resulted in improvements in FEV_1_ (10%), sweat chloride (−45.1 mmol/L) and CFQ-R score (17.4 points). The 4-week trial did not reveal any changes in the rate of pulmonary exacerbations, although the 24-week data from subjects with the F508del/MF genotype strongly suggest that a decrease in the rate of pulmonary exacerbations is also to be expected over longer periods of time in subjects with the F508del homozygous genotype.

ELX/TEZ/IVA has now been studied in children 6–11 years with at least one F508del allele and shown to be well-tolerated and safe [94]. Sixteen children received therapy for 2 weeks for pharmacokinetic, efficacy and safety studies. Efficacy and safety data in 66 children having received at least one dose were further monitored over 24 weeks. The doses for children who weighed less than 30 kg were 50% of the adult dose (elexacaftor 100 mg qd, tezacaftor 50 mg qd, ivacaftor 75 mg q12h), whereas children weighing 30 kg or more received the usual adult dose. Marked improvements were reported for sweat chloride concentration (−60.9 mmol/L), ppFEV_1_ (10.2%), lung clearance index (LCI −1.7 units), CFQ-R respiratory domain score (7 points) and body mass index for age z-score at 24 weeks. Liver aminotransferase increases threefold above the upper limit of normal for age were observed in 10.6% of patients. 

ELX/TEZ/IVA has also been shown to be safe and effective in patients with at least one F508del mutation and severe CF lung disease, defined as a ppFEV_1_ below 40% [95,96]. Therapy also decreases sinonasal symptoms [97].

While the elexacaftor component of ELX/TEZ/IVA clearly has corrector function, it also has potentiator activity. Elexacaftor combined with ivacaftor markedly improves CFTR gating function, suggesting that ivacaftor and elexacaftor dual therapy may be even more effective in patients with the G551D or other dual potentiator responsive mutations [98].

#### 2.2.2. Other Modulators in the Pipeline

Other CFTR potentiators and correctors are currently in the pre-clinical or clinical phases of development, as summarized in recent reviews and listed in Table 2 [3,64,99].

#### 2.2.3. Personalized Medicine

Clinical development programs of new therapies for people with CF have revealed marked differences in individual responses to therapies, thus making it difficult to choose the best therapy for some individuals. Although the *CFTR* genotype is a major factor in defining therapeutic responses, genetic and environmental factors independent of *CFTR* alleles markedly affect responses to modulator therapy. There is a clear need for diagnostic tests (theranostics) to better predict treatment responses to modulators for each CF individual particularly in the presence of rare *CFTR* mutations. Several predictive outcome measures are proposed to monitor individual responses, including ex vivo testing of rare mutation response to modulators in Fisher rat thyroid cells for class III mutations [60], sweat chloride concentration [100], induced pluripotent cells [101] and intestinal organoids [102,103,104]. Theranostics for CF have been reviewed by the European Cystic Fibrosis Society strategic planning task force on ‘Speeding up access to new drugs for CF’ [105]. Among these assays, the intestinal organoids show particular promise for adapting new therapies to individual needs. CFTR functional response to CFTR modulator therapy is reflected by ex vivo measurements of intestinal organoid forskolin-induced swelling (FIS) that have been shown to closely correlate with long-term clinical responses, making this an attractive assay to predict individual responses to modulators in patients carrying rare *CFTR* mutations [102,106,107,108]. An alternate approach is the CF Canada Sick Kids Program for Individualized CF Therapy (CFIT) in which nasal cells, blood cell-derived induced pluripotent stem cells, gene expression data and clinical data are collected from CF patients to help predict individual responses to new therapies [101].

### 2.3. CFTR Amplifiers

A novel therapeutic strategy independent of the *CFTR* mutation class is to amplify the mRNA and allow increased translation of protein. The CFTR protein can then be rescued in conjunction with correctors and potentiators to increase in CFTR protein density and function at the cell’s apical surface [109]. Amplifiers increase mRNA translation and decrease mRNA decay by facilitating the transition of nascent polypeptides through a protein complex known as the translocon to the endoplasmic reticulum for integration into the membrane.

In a phase II clinical trial of 24 patients with CF and the F508del homozygous genotype all receiving LUM/IVA therapy, the amplifier PTI-428 (nesolicaftor) at 50 mg id was compared to placebo (randomized active:placebo 4:1) over 28 days [110]. The ppFEV_1_ of the group receiving active drug increased by 5.2% over days 14–28 and the therapy was well-tolerated; however, no further clinical studies of this compound are currently planned.

### 2.4. Pre-Termination Codon Agents

Currently, 10% of individuals with classical CF cannot benefit from any of the CFTR modulators that have been approved for clinical use. Many of these individuals express rare mutations of class IA or IB or for other reasons do not respond to potentiators and correctors. The majority of these individuals have at least one allele with a pre-termination codon (PTC), a defect found in 11% of all CF disease-causing alleles. Strategies to address PTC defects include read-through agents with CFTR modulators, compounds that reduce NMD, gene editing to correct PTC non-sense mutations and genetic therapies.

Prior to the development of CFTR modulators, initial in vitro observations with the aminoglycoside antibiotic gentamicin revealed that it is possible to induce miscoding errors in the ribosome and suppress the premature termination of translation encoded by PTCs [111]. However, increasing the read-through of PTC is not always as efficient as expected since NMD varies considerably between cells [12]. NMD occurs once SMG-1 phosphorylates the Upf1 protein, which then becomes an active helicase that degrades mRNA. While NMD markedly decreases mRNA and protein stability in cells with a *CFTR* class I mutation [112], it is possible to inhibit the NMD factor SMG-1 using antisense oligonucleotides to increase the mRNA, protein and cell surface expression of W1282X-CFTR [113].

The discovery of the small molecule PTC124 (ataluren), which allows the ribosomal machinery to read through some PTC alleles, provided hope for many persons with CF. Early phase II clinical trials results of ataluren were encouraging, but a phase III 48-week double-blind placebo controlled clinical trial showed no benefit in the primary outcome, ppFEV_1_ [114]. Since individuals within the study who were not receiving inhaled aminoglycosides seemed to show some benefit, a second phase III trial was conducted that excluded the use in inhaled aminoglycosides. Yet again, the trial failed and the ataluren development program in CF has since been stopped.

More recently, an open-label dose escalation study of ELX-02, a eukaryotic ribosomal selective glycoside given subcutaneously, has been initiated in CF patients with a G542X/MF genotype to assess safety, tolerability, pharmacokinetics and pharmacodynamics. Secondary outcomes will include lung function assessments over 9 weeks (Table 2 for trial registration). Previous studies using intestinal patient-derived organoids (PDO) with the G542X/G542X, G542X/W1282X or G542X/MF genotypes showed significant restoration of CFTR-dependent PDO swelling [115]. Interestingly, ELX-02 may also reduce NMD, thus increasing the hope that it may help restore CFTR protein [116].

Further evidence suggests that combination of CFTR correctors, potentiators and PTC read-through agents represents a promising strategy that provides superior restoration of CFTR function when compared to CFTR modulators or nonsense mutation agents alone [117].

### 2.5. CFTR Stabilizers

Misfolded CFTR proteins such as F508del inserted into the plasma membrane is at risk of removal by the peripheral protein quality control system. Cytoplasmic regions of unfolded CFTR are ubiquitinated and removed from the plasma membrane by endocytosis and lysosomal degradation [118]. The CFTR stabilizers are agents that interfere with the plasma membrane protein quality control process. Among the stabilizers is hepatocyte growth factor (HGF), which activates Rac1 signaling and anchors CFTR to the apical actin cytoskeleton via ezrin, thus helping to maintain localization of misfolded F508del CFTR in the plasma membrane [119].

Vasoactive intestinal peptide (VIP) increases cAMP and thus stimulates CFTR-dependent chloride secretion. VIP also stabilizes CFTR at the plasma membrane by enhancing the phosphorylation of the actin-binding complex ezrin/radixin/moesin (ERM) which interacts with the scaffolding protein Na^+^/H^+^ exchange factor1 (NHERF-1) and CFTR to prevent CFTR endocytosis and lysosomal degradation [120].

Cavosonstat (N91115) is an S-nitrosoglutathione (GSNO) reductase inhibitor. Inhibition of GSNO reductase increases cellular GSNO which can s-nitrosylate the Hsp70/Hsp90 organizing protein (HOP) and stabilize cell surface expression of F508del CFTR [121]. Cavosonstat has been the object of phase I clinical trials [122] in CF patients; however, a phase II trial of cavosonstat in combination with ivacaftor did not meet the primary endpoint of a change in lung function, and currently there is no further development of this compound.

### 2.6. Genetic Therapies

Technologies aimed at the delivery of mRNA to humans have rapidly gained interest in the therapeutics and vaccine fields. Nanoparticles comprised of ionizable lipid, cholesterol, phospholipid and ethylene glycol have been engineered to deliver mRNA using membrane destabilization and endosomal escape while evading host immune defenses. Successful delivery of mRNA and production of functional CFTR protein has been demonstrated in a murine model of CF in which aerosolization of nanoparticles carrying *CFTR* mRNA induced the expression of sufficient CFTR protein to restore iodide efflux from CF airway cells up to 55% of that observed in healthy mice 3 days after transfection [123]. The level of CFTR functional restoration in treated CF mice approximated that achieved by ivacaftor.

Translate Bio is currently conducting a phase I/II first in human randomized placebo-controlled trial with multiple escalating doses to test the safety and tolerability of MRT5005 nanoparticles to deliver *CFTR* mRNA to the airways in CF adults (NCT03375047). The change in ppFEV_1_ will also be assessed as a secondary outcome.

An alternate approach to deliver functional mRNA and protein to the airway is gene therapy. Although considered as a promising therapy since the discovery of the *CFTR* gene over thirty years ago, CF gene therapy development has proven quite challenging since vectors must have sufficient carrying capacity, express a capsid with tropism for the human airway and be of low immunogenicity. The low immunogenicity and airway cell tropism of the adeno-associated viral vector (AAV) would seem to make this an ideal vector, but the AAV vector has limited carrying capacity. The CF gene should also integrate into the host genome to avoid repeated administration of the vector. Using specific nucleotide modifications tailored to meet each of these requirements, Cooney et al. designed a shorter *CFTR* gene in which some nucleotides of the *CFTR* R domain were removed, a minimal number of terminal repeats (TR) of the *piggyBac* transposon were added and a short promoter and polyadenylation signal was used to produce a nucleotide sequence that fits into the AAV vector [124]. Aerosol delivery of the PB/AAV^CFTR∆R^ in gut-corrected CF pigs resulted in correction of airway *CFTR* expression, transepithelial current, airway surface liquid pH, mucus viscosity and bacterial killing, thus providing proof that aerosol delivery of the shortened *CFTR* using the PB/AAV^CFTR∆^ vector can correct the CF phenotype in airways. The addition of transposase to the PB/AAV^CFTR∆^ transfection system in vitro in HeLa cells resulted in persistent *CFTR* expression. Although major challenges such as effective in vivo delivery to appropriate airways cells remain, these results raise hopes of a single-dose lifelong treatment for CF lung disease through integration of the curative transposon in a safe harbor of the host genome [125].

Another approach to permanent therapeutic gene expression is through the use of site-specific endonucleases such as clustered regularly interspaced short palindromic repeats (CRISPR-Cas9). The CRISPR-Cas9 system allows specific guide RNA sequences to target precise locations within the genome [126] such as the *GGTA1* locus, a known safe harbor for exogenous transgene expression. To test the feasibility of this approach, Zhou et al. used the helper-dependent adenoviral (HD-Ad) vector HD-Ad-Cas9-hCFTR to deliver the *hCFTR* gene for integration at the *GGTA1* locus in porcine *CFTR* knockout cell line [127]. Not only did *CFTR* expression persist after many passages, but viral genome copy numbers were greatly reduced with passage of cells resulting in the desired outcome of persistent therapeutic gene expression and disappearance of traces of viral genetic material. While much still needs to be done, improved technologies for efficient safe and persistent *CFTR* gene delivery are rekindling hopes of translating gene therapy into clinical applications for CF patients.

## 3. Alternate Ion Channel Modulation

### 3.1. ENaC Inhibitors

The epithelial sodium channel (ENaC) located at the apical membrane of airway cells is the key regulator of sodium absorption, determining the degree of water absorption through the paracellular shunt pathway. Together with CFTR, ENaC dictates airway surface liquid hydration [128]. When CFTR is dysfunctional, ENaC activity is increased [129] and the ASL height is low, mucus solids increase, and ciliary beat frequency is decreased. The absence of functional CFTR-dependent chloride secretion combined with the increase in ENaC activity compounds the mucociliary clearance defect causing CF [130]. Therefore, ENaC inhibitors constitute attractive potential therapeutic agents for all individuals with CF regardless of their *CFTR* genotype. Furthermore, intracellular and extracellular proteases at the airway surface are needed to activate ENaC and constitute another potential therapeutic target to regulate ASL hydration. Pre-clinical studies using CFTR-deficient cells and mice have confirmed the importance of ENaC in regulating the volume of ASL [131]. These encouraging results led to several clinical trials that target ENaC in CF patients.

Clinical trials to develop small molecules that regulate ENaC have been disappointing [132]. Some programs were discontinued in phase I due to acute hyperkalemia (GS-9411) or palatability issues (BI 443651). Several other programs have been discontinued in phase II due to the lack of efficacy such as those of the direct ENaC inhibitors amiloride and VX-371, the SPLUNC-1 analogue SPX-101 and the protease inhibitor camostat [132]. However, the small molecule BI 1265162 is a potent direct ENaC inhibitor that did not induce hyperkalemia, was well-tolerated in phase I studies and is currently the object of a phase II clinical trial [133,134].

### 3.2. Calcium-Activated Chloride Secretion and TMEM16A Potentiator

TMEM16A is a calcium-activated chloride channel expressed at the apical surface of respiratory epithelial cells as well as in goblet cells, smooth muscle and neuronal cells [135]. Chloride conductance of TMEM16A can be indirectly stimulated by molecules that increase cytoplasmic calcium such as the P_2_Y_2_ purine receptor agonist denufosol or the calcium ionophore duramycin (Moli-1901) [136,137]. After initial results of a phase III trial of denufosol showed a statistically significant but modest improvement in ppFEV_1_ at 24 weeks [138], a second phase III trial (TIGER-2) failed to show benefit [139], and development of denufosol was stopped. Furthermore, initial results of a phase II clinical trial with inhaled Moli-1901in CF indicated significant improvement in ppFEV_1_ in a small number of CF patients, but there has been no further clinical development [140]. Recently, a direct potentiator of TMEM16A, ETX001 (Enterprise Therapeutics) has been reported to increase ASL volume in cells from patients homozygous for F508del [141]. Inhaled ETX001 accelerated tracheal mucus velocity and mucociliary clearance in an ovine model with and without chemical suppression of respiratory CFTR using inhaled CFTR_Inh172_ [141]. These results support the exploration of TMEM16A potentiators as a possible new treatment of the CF basic defect. Clinical studies of EDT002, a first-in-man TMEM16A potentiator, are planned (NCT04488705) and will necessarily include safety and tolerability considerations since TMEM16A is expressed in many tissues other than those primarily affected in CF [142].

### 3.3. Amphotericin

Amphotericin B (AmB) is an antifungal medication and small molecule that forms monovalent ion channels unselective for anions and cations. Anion secretion through the apical membrane of respiratory epithelial cells is dependent upon an electrochemical gradient generated by Na^+^/K^+^ ATPase pump and the Na^+^/K^+^/2Cl^−^ co-transporter situated in the cell’s basolateral membrane. The addition of AmB to cell lines and primary airway epithelial cells with defective CFTR restored apical membrane bicarbonate secretion and increased ASL volume [143]. AmB-induced bicarbonate secretion was inhibited by ouabain, an inhibitor of the Na^+^/K^+^ ATPase pump, and inhibition of the Na^+^/K^+^/2Cl^−^ co-transporter prevented the AmB-induced increase in ASL height, suggesting the importance of basolateral proteins and K^+^ flux in driving anion secretion at the apical membrane. Primary cultures of polarized epithelial cells derived from donors with CF and different *CFTR* mutations treated with AmB also demonstrated correction of the ASL pH and bacterial killing activity, and the addition of AmB to the tracheal of the CFTR^−/−^ pig increased ASL pH.

Since AmB is approved for clinical use and has a known safety profile, these exciting results have led to a rapid translation into early clinical trials in people with CF. The addition of AmB to the nasal mucosa of CF individuals not on modulator therapy with various genotypes resulted in significant correction of the nasal potential differences in the direction and of the magnitude observed with ivacaftor treatment in people with CF and a G551D mutation [144].

### 3.4. Anionophores

Anionophores are small molecule synthetic anion carriers which can restore transmembrane ion conductance at concentrations that are non-toxic [145]. Several anionophores at non-toxic concentrations have been shown to restore transmembrane halide flux in cells without CFTR [146,147,148]. Two anionophores with structures akin to the natural products prodiginines and tambjamines, MM3 and MM34 added to human bronchial epithelial cell monolayers carrying the homozygous F508del genotype have been shown to restore transepithelial electrical conductance, increase ASL pH, prevent fluid re-absorption and decrease ASL viscosity [149]. Anionophores represent a novel class of small molecules with the potential to correct the basic CF-causing defect independently of *CFTR* genotype and raise hopes of future clinical trials for people with CF, regardless of their genotype.

### 3.5. Antisense Oligonucleotides

Antisense oligonucleotides (ASO) are short single strands of nucleotides that hybridize to complementary RNA sequences through base-pairing and induce a variety of actions on the target RNA that can be modulated according to the pharmacologic goal [150]. The RNase H1 enzyme cleaves target RNA that is in a DNA-RNA heteroduplex such as occurs with the binding of ASO to target RNA [151]. The subsequent degradation or modification of target RNA can result in therapeutic activity relevant to CF such as inhibition of NMD or decrease in ENaC activity [113,152]. Another potential therapeutic benefit of ASO technology for CF includes its capacity to alter RNA splicing. Splice-switching ASO targeting the *CFTR* 3849 + 10 kb C > T mutation has recently been shown to restore chloride secretion in bronchial epithelial cells [153].

## 4. Conclusions

The therapeutic landscape for CF individuals has changed dramatically in a very short time. Up to 90% of CF patients are eligible for highly effective CFTR modulator therapies and great strides are being made to address the basic CF defect in the remaining 10%. Despite these encouraging steps, many CF patients worldwide do not have access to highly effective CFTR modulator therapy and almost all patients have the same treatment burden as before. There is hope that the treatment burden can be decreased using highly effective CFTR modulator therapy, a goal that is currently being explored by investigators of the SIMPLIFY protocol [154]. If new therapies allow a decreased use of burdensome treatments, then this will represent a major step forward in improving the quality of life for CF patients. However, in addition to the challenges of decreasing treatment burden, and finding effective therapies for all individuals with CF using personalized medicine, the issue of access to costly but highly effective treatments for all patients urgently needs to be addressed. Meeting this goal may represent the greatest hurdle of all in the field of new therapies for persons with CF. Hopefully, the enormous creativity, dedication and resolve of scientists, caregivers, patients and industry partners will find ways to meet this challenge sooner than later.

## Figures and Tables

**Table 1:** FDA-approved CFTR modulators and indications for use in CF individuals.

Drug (Trade Name)	Mode of Action	Age	Mutation Class	Alleles
Ivacaftor (Kalydeko)	Potentiator	2 years and older	III, IV	*G551D, G1244E, G1349D, G178R, G551S, S1251N, S1255P, S549N, S549R, R117H*, other rare mutations (see text)
Ivacaftor and lumacaftor (Orkambi)	Potentiator and corrector	12 years and older	II	*F508del* homozygous
Ivacaftor and tezacaftor (Symdeko)	Potentiator and second-generation corrector	12 years and older	II/II, or II/RF *	*F508del* homozygous or *F508del/RF* *
Ivacaftor, tezacaftor and elexacaftor (Trikafta)	Potentiator and second-generation corrector and next-generation corrector	12 years and older	II/II, II/other	A diagnosis of CF and at least one *F508del* OR another *CFTR* responsive mutation **

* RF, residual function *CFTR* mutation including: E56K, P67L, R74W, D110E, D110H, R117C, E193K, L206W, R347H, R352Q, A455E, D579G, 711 + 3A→G, E831X, S945L, S977F, F1052V, K1060T, A1067T, R1070W, F1074L, D1152H, D1270N, 2789 + 5G→A, 3272−26A→G, 3849 + 10kbC→T. ** a mutation in the CFTR gene that is responsive based on CFTR chloride transport assay in Fisher rat thyroid cells expressing mutant CFTR.

**Table 2:** CFTR modulators other than those in current clinical use.

Molecule ID	Company	Mechanism	Clinical Trial Number
VX-561 deutivacaftor	Vertex Pharmaceuticals	Potentiator	NCT03911713
ABBV-974	AbbVie	Potentiator	NCT02707562 NCT02690519
ABBV-2451	AbbVie	Potentiator	NCT03540524
ABBC-3067	AbbVie	Potentiator	NCT03969888
QBW251 icenticaftor	Novartis	Potentiator	NCT02190604
VX-121	Vertex Pharmaceuticals	Corrector	NCT03912233 NCT03768089
VX-440 olacaftor	Vertex Pharmaceuticals	Corrector	NCT02951182
VX-659 bamocaftor	Vertex Pharmaceuticals	Corrector	NCT03447249
ABBV-2222 galicaftor	AbbVie	Corrector	NCT03969888
ELX-02	Eloxx Pharmaceuticals	Read-through	NCT04135495 NCT04126473

## Data Availability

Not applicable.
